# Backward bifurcations and strong Allee effects in matrix models for the dynamics of structured populations

**DOI:** 10.1080/17513758.2014.899638

**Published:** 2014-03-31

**Authors:** J.M. Cushing

**Affiliations:** ^a^ Department of Mathematics, University of Arizona, 617 N Santa Rita, Tucson, AZ85721, USA; ^b^ Interdisciplinary Program in Applied Mathematics, University of Arizona, Tucson, AZ85721, USA

**Keywords:** Allee effects, structured population dynamics, equilibrium, bifurcation, backward bifurcation, stability

## Abstract

In nonlinear matrix models, strong Allee effects typically arise when the fundamental bifurcation of positive equilibria from the extinction equilibrium at *r*=1 (or *R*_0_=1) is backward. This occurs when positive feedback (component Allee) effects are dominant at low densities and negative feedback effects are dominant at high densities. This scenario allows population survival when *r* (or equivalently *R*_0_) is *less* than 1, provided population densities are sufficiently high. For *r*>1 (or equivalently *R*_0_>1) the extinction equilibrium is unstable and a strong Allee effect cannot occur. We give criteria sufficient for a strong Allee effect to occur in a general nonlinear matrix model. A juvenile–adult example model illustrates the criteria as well as some other possible phenomena concerning strong Allee effects (such as positive cycles instead of equilibria).

## 1. Introduction

The majority of nonlinear population dynamic models appearing in the literature are based on negative feedback effects caused by increased population density. That is to say, they are based on negative correlations between increased population density and (per capita) model components that describe vital parameters relating to individual fitness (fertility, survivorship, growth rates, metabolic rates, etc.). Although interest in positive correlations between increased density and fitness arose as early as the 1930s in the work of Allee [1–3], little theoretical or applied studies of such effects appeared in the literature until the end of last century. A seminal paper of Dennis [18] describes the early history of Allee effects and introduces some basic models in which fertility (specifically, the probability of finding mates) is the mechanism responsible for the positive feedback effects. In his work, Allee described many other biological and environmental causes that can be responsible for such positive feedback effects. The recent book by Courchamp *et al.* [5] also describes many mechanisms for Allee effects that have now been well documented by ecologists. This book and its extensive bibliography demonstrate the exponential growth of interest in Allee effects that has occurred during the last couple of decades. This interest has been spurred by concerns about endangered species, conservation management, sustainability, species extinction, and biological diversity. Positive feedback effects, due to increases in low level densities, on parameters relating to individual fitness – called *component Allee effects* – can, if sufficiently ‘strong’, lead to a threshold population density below which a population will go extinct. It is argued that this threshold can be significantly higher than that at which random events dominate extinction events and therefore provide a deterministic mechanism for extinction [5,18].

We say that a population dynamic model has a *strong Allee effect* if there exist both a positive and an extinction attractor. The typical case involves equilibria, i.e. an extinction equilibrium and a positive equilibrium, both of which are stable. (However, we give an example in Section 4 in which a strong Allee effect occurs with a stable positive 2-cycle in place of a stable positive equilibrium.) In this paper, strong Allee effects are considered from the point of view of bifurcation theory and their relation to a backward bifurcation is explored. Because strong Allee effects concern extinction, the stability or instability of an extinction equilibrium in a model is of fundamental interest. The stability of the extinction equilibrium is, of course, governed by the parameter values in the model. In general, convenient parameters for such an investigation are the inherent population growth rate *r* and the inherent net reproductive number *R*_0_. These quantities (defined for matrix models in Section 2) are combinations of model parameters related to various vital rates (birth, death, growth, maturation, etc.). The extinction equilibrium is stable if *r*<1 (equivalently *R*_0_<1) and is unstable if *r*>1 (equivalently *R*_0_>1). Therefore, in general, a strong Allee effect cannot occur if *r*>1 (or equivalently if *R*_0_>1).

In this paper, a bifurcation theoretical approach is taken to study strong Allee effects in nonlinear matrix models for the discrete time dynamics of structured populations [4,12,14]. Theorem 3 in Section 3 gives criteria sufficient to guarantee that a strong Allee effect occurs in a nonlinear matrix model for values of *r*<1 (or *R*_0_<1). Key to this approach are the fundamental bifurcation theorems 1 and 2 in Section 2. These theorems describe how the destabilization of the extinction equilibrium at *r*=1 (*R*_0_=1) results in the bifurcation, from the extinction equilibrium, of a continuum of positive (survival) equilibria and how, at least near bifurcation, the stability of these bifurcating positive equilibria depends on the direction of bifurcation. Forward bifurcating positive equilibria (which correspond to 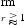 or 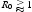) are stable while backward bifurcating equilibria (which correspond to 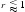 or 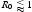) are unstable. As seen in Section 2, backward bifurcations occur when the positive feedbacks, arising from the model's component Allee effects at low densities, are sufficiently large in magnitude (relative to any negative feedback effects due to increased low-level density). It will turn out that this low density phenomenon, when coupled with dominant negative feedbacks at high population levels, can lead to strong Allee effects. Mathematically, this occurs because the backward bifurcating continuum ‘folds over’ or ‘turns around’ at a saddle-node bifurcation (sometimes called a blue-sky or tangent bifurcation) and creates multiple positive (survival) equilibria, which in turn can, if they are stable, create a strong Allee effect.

We focus on strong Allee effects involving a stable extinction equilibrium in the presence of a stable positive (survival) equilibrium, as opposed to other kinds of positive attractors. However, the example given in Section 4 not only demonstrates the theorems of Section 3, but also provides an example of a strong Allee effect associated with a stable positive cycle rather than a stable positive equilibrium.

## 2. A fundamental bifurcation theorem for matrix models

Let 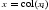 denote a column vector in *n*-dimensional Euclidean space *R* ^*n*^ and let

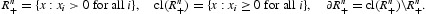



Let Ω be an open set in *R* ^*n*^ such that 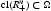. We denote the spectral radius of an *n*×*n* matrix *M* by ρ[*M*] and use the norm

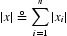

on *R* ^*n*^.

The vector *x* represents a distribution of population densities in *n*-classes which we wish to follow in time. The discrete time dynamics are described by the equation

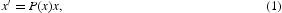

where 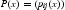 is an *n*×*n* matrix and the prime ‘^′^’ denotes the distribution vector at the next time step [21]. We assume

A1: 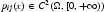 and *P*(*x*) is primitive for all *x*∈Ω.

Under A1, we know from Perron–Frobenius theory that for each *x*∈Ω the matrix *P*(*x*)

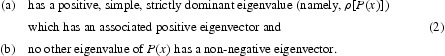

In order to introduce a bifurcation parameter into Equation (1) we write

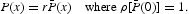

Note that (a) and (b) are also true for the normalized projection matrix 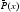. The quantity 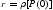, which is the strictly dominant eigenvalue of *P*(0), is the *inherent population growth rate*. We re-write Equation (1) as

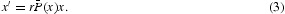

An equilibrium (fixed point) *x*∈*R* ^*n*^ of Equation (3) satisfies the algebraic equation

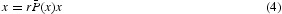

and, following [24], we refer to 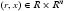 as an *equilibrium pair*. If a solution of Equation (4) lies in 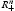 we say it is a *positive* equilibrium, in which case we say 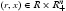 is a *positive equilibrium pair*.

Write the equilibrium equation (4) as

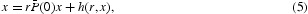

where

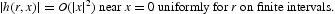



The Rabinowitz alternative from nonlinear functional analysis [24, Theorems 1.3 and 1.40] guarantees the existence of *a (maximal) continuum* 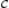 *of equilibrium pairs* of Equation (5) that bifurcates from (*r, x*)=(1, 0) (i.e. a closed and connected set of equilibrium pairs that contains (1, 0)) for which 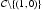 lies in 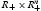 (i.e. consists positive equilibrium pairs) near (*r, x*)=(1, 0) and which either (i) meets infinity (i.e. is unbounded) in 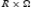 or (ii) meets (*rˆ*, 0) where *rˆ*≠1 is a real characteristic value of 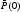.^1^ Also see [20]. For a proof of the following result, see Appendix 1.

Theorem 1 Under assumption *A1*, the matrix Equation (4) has an unbounded continuum 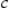 of equilibrium pairs that bifurcates from (*r, x*)=(1, 0) and for which 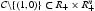.

This theorem says, under assumption A1, that the matrix equation (4) has an unbounded continuum of positive solution pairs that bifurcates from (*r, x*)=(1, 0). Let

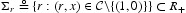

denote the *spectrum of* 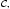 i.e. the set of *r* values corresponding to positive equilibria from the continuum 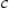. Let the *range of* 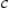 be denoted by

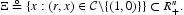

The range Ξ is the set of positive equilibria associated with the continuum 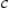. Since 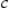 is a continuum, the closures 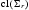 and cl(Ξ) of Σ_*r*_ and Ξ in 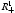 and 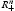, respectively, are continua. Moreover, it follows from Theorem 1 that 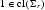 and either the spectrum or the range (or both) are unbounded.

The bifurcation occurring at (*r, x*)=(1, 0) in Theorem 1 is said to be *forward* (or supercritical or to-the-right) if *r*>1 for those equilibrium pairs 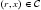 in a neighbourhood of (1, 0). If, on the other hand, *r*<1 for equilibrium pairs 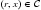 in a neighbourhood of (1, 0), then the bifurcation is said to be *backward* (or subcritical or to-the-left).

The next theorem shows that the stability of the equilibria lying on the bifurcating continuum 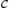 near the bifurcation point (1, 0) is determined by the direction of bifurcation, which in turn is determined by sign the quantity

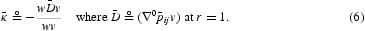

Here, *v*>0 and *w*>0 are the right and left eigenvectors associated with eigenvalue 1 of 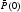 and 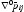 is the gradient of 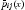 with respect to *x* evaluated at *x*=0 (written as a row vector). Since 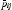 is a scalar multiple *r* of *p*_*ij*_, the sign of 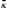 is the same as the sign of

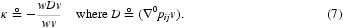

Theorem 2 [12,14] Assume *A1*. Near the point (*r, x*)=(1, 0), we have the following two alternatives.

(a) κ<0 implies the bifurcation of 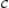 is backward and the positive equilibria *x*∈Ξ from the continuum 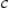 are unstable for 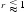.
(b)  κ>0 implies the bifurcation of 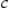 forward and the positive equilibria *x*∈Ξ from the continuum 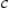 are (locally asymptotically) stable for 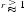.

*Note 1.* If all partial derivatives of entries in the projection matrix satisfy 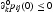 and are not all equal 0, then κ>0 and the bifurcation is forward and hence stable. This is a useful observation since in applications one can often observe this criterion by inspection and avoid the calculation of κ or any linearized stability analysis.

For κ<0 to hold, at least one derivative 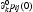 must be positive. That is to say, at least one component in the projection matrix must have a positive feedback relationship with increased density in some demographic class, i.e. a component Allee effect. Such component Allee effects do not necessarily lead to a backward bifurcation, however. To do so, their magnitudes must be sufficiently large so as to dominate any negative derivatives appearing in κ and make κ negative. This would certainly be true, for example, if all partial derivatives satisfy 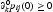 and all are not equal to 0, that is to say, if there are no low density negative feedback components at all (and at least one component Allee effect is present).

*Note 2.* Given the generality of Theorems 1 and 2 (and their analogous counterparts for other types of population models, such as models based on ordinary and partial differential, integral, integro-differential, integro-difference, etc.), they can together be referred to as a fundamental bifurcation theorem for nonlinear population dynamics.

## 3. Strong Allee effects from backward bifurcations

In this section, we consider the matrix equation (3) when it has a backward bifurcation at (*r, x*)=(1, 0). Therefore, we assume

A2: κ<0 where κ is given by Equation (7).

Under this assumption the matrix equation (3) has, by Theorem 2, a backward bifurcation at *r*=1 and consequently has unstable positive equilibria for 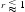. Recall that the extinction equilibrium *x*=0 is stable when *r*<1. In addition, we make the following assumption.

A3: (a) there exists an 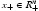 such that 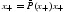; (b) 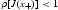.

Another way of stating A3 is that (a) there exists a positive equilibrium 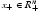 of the matrix equation (1) with *r*=1 and (b) this positive equilibrium is (locally asymptotically) stable by linearization.

Theorem 3 Under assumptions *A1, A2* and *A3* matrix equation (3) has a strong Allee effect at least for 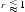.

For a proof of this theorem, see Appendix 2.

*Note 3.* For the case *n*=1 of one-dimensional maps, Theorem 3 provides criteria that are sufficient for the occurrence of a strong Allee effect involving a stable positive and extinction equilibria. These criteria, which are sufficient (but not necessary) for a strong Allee effect by our definition, are the same criteria used in [23] to define a strong Allee effect in an one-dimensional map.

One way to guarantee A3(a) holds is to show two things: that the bifurcation is backward (for example by calculating κ or 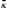) and that the spectrum Σ_*r*_ is unbounded in *R*_+_. From these facts it follows that 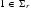, which implies in turn that A3(a) holds.

In applications, one can often show the spectrum Σ_*r*_ is unbounded by an investigation of the equilibrium equation

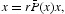

which, of course, is satisfied by all pairs 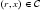. For example, consider the following assumption.

A4: There exists a real-valued function *m*(*r*), defined for *r*≥1 and bounded on bounded subintervals, such that for every equilibrium pair (*r, x*)∈ 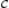 the inequality

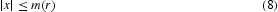

holds.

Under the a priori bound (8) the spectrum Σ_*r*_ is unbounded (because a bounded spectrum would imply a bounded range, which would contradict the fact that the continuum 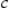 is unbounded).

Corollary 1 Suppose *A1* and *A4* hold. The spectrum Σ_*r*_ is unbounded and hence 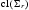 is an interval of the form 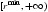 for 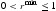.

If the spectrum is unbounded and a backward bifurcation occurs, then *r* ^min^<1. It follows that 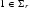, which implies that there is a positive equilibrium corresponding to *r*=1.

Corollary 2 If *A1, A2* and *A4* hold, then 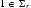 and A3(a) holds.

*Note 4.* It is, of course, possible that A3(a) holds but A3(b) fails to hold. We will see an example of this possibility in Section 4.

*Note 5.* We have assumed that the projection matrix *P*(*x*) in the matrix equation (1) has the form:

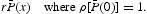

This normalization implies the coefficient *r* is the spectral radius of the projection matrix at *x*=0 and is therefore the inherent population growth rate. This normalization is convenient for theoretical purposes, but in applications it is unlikely possible to write the normalization explicitly since formulas for *r* are not in general available. Instead, it is often more convenient to use the inherent net reproductive number *R*_0_ as the bifurcation parameter, which as it turns out can be substituted for *r* in the theorems above. The quantity *R*_0_ is defined as follows.

In models of populations closed to immigration/emigration, the projection matrix can be additively decomposed

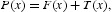

where *F*(*x*) is a non-negative matrix consisting of class-specific fecundities (that is to say, the distribution newborns at the next time step is *F*(*x*) *x*). The transition matrix *T*(*x*) describes how surviving individuals move between classes. Specifically, the *ij*th entry in *T*(*x*) is the fraction of *j*-class individuals that survive (a unit of time) and move to class *j*. The transition matrix *T*(*x*) is therefore non-negative, has entries that are less than or equal to 1 and column sums less than or equal to 1. The inherent net reproductive number is defined to be

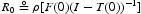

(where *I* is the *n*×*n* identity matrix). If 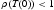 (which means the expected life time of an individual is finite), then it is known that *r* and *R*_0_ satisfy one of the following [14,17,22]:

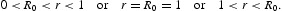



The equilibrium equation *x*=*P*(*x*)*x* can be replaced by

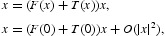

and, if we normalize the fertility matrix so that 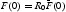 where 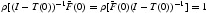, by

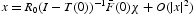

and equation that can be analysed by the Rabinowitz alternatives using *R*_0_ as the bifurcation parameter. For these reasons,

*r* can be replaced by *R*_0_ in assumption A4, Theorems 1–3, and Corollaries 1 and 2. The spectrum 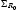 of *R*_0_ values from the continuum *C* of positive equilibrium pairs (*R*_0_, *x*) replaces that of the spectrum of *r* values.

The advantage of using *R*_0_ as the bifurcation parameter is that it is often the case that explicit formulas in terms of the entries in the projection matrix *P* are available for *R*_0_, but not for *r* [12,14]. We will use this fact in the next section.

## 4. An example

The 2×2 projection matrix

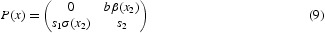

in the matrix model (1) describes a population classified into juveniles *x*_1_ and adults *x*_2_. It assumes that the density effects occur in juvenile survival 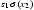 and adult fecundity *b*β(*x*_2_) and depend only on adult density *x*_2_. Adult survivorship *s*_2_ is assumed density independent.

We assume that increased adult density *x*_2_, at all levels, has a negative effect on juvenile survival so that σ(*x*_2_) is a decreasing function of *x*_2_. On the other hand, we assume that increased adult density *x*_2_, at least at low densities, has a *positive* effect on adult fecundity so that β(*x*_2_) is an increasing function of 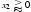. Under this assumption *b*β(*x*_2_) entails a component Allee effect.

Specifically, we assume the entries in the projection matrix (9) satisfy the following conditions:

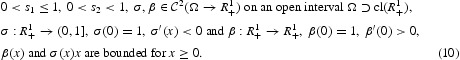

We apply Theorem 3 using the inherent net reproductive number in place of *r* (see Note 5)

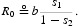

To do this we need to investigate the assumptions A2 and A3 required for that theorem.

Right and left eigenvectors *v* and *w* associated with eigenvalue 1 of *P*(0) when *R*_0_=1, i.e. of the matrix

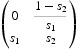

are

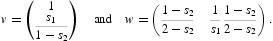

These are chosen so that *wv*=1 and hence 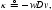 where

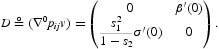

Consequently,

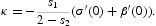

Assumption A2 holds and a backward bifurcation occurs if

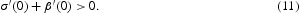

This inequality is interpreted as meaning that the positive feedback effect of adult density on adult fecundity, as measured by 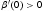, outweighs its negative effect on juvenile survival, as measured by 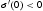.

To investigate A3(a) we re-write the equilibrium equations

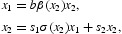

equivalently as

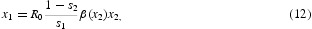

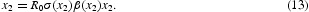

For any positive solution of these equations we have, by Equations (10) and (12b), the bound 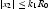 for some constant *k*_1_>0. From Equation (12a) we obtain, for some constant *k*_2_>0 such that 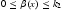, the bound

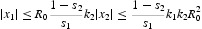

and hence the bound

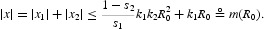



The requirement A4 (with *R*_0_ replacing *r*, as in Note 5) is satisfied. Corollaries 1 and 2 imply that A3(a) holds, i.e. that there exists a positive equilibrium 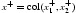 with *R*_0_=1.

Since the product 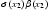 equals 1 and has a positive derivative at *x*_2_=0 and since it also equals 1 when evaluated at a positive 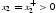, it follows that the equation 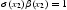 has a (at least one) positive root 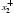 at which 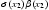 is non-increasing. We assume something slightly stronger, namely, that there is a positive root of 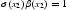 at which 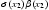 strictly decreasing, i.e.

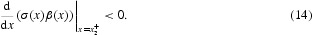

Finally, we need to consider assumption A3(b), namely the requirement that the positive equilibrium

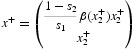

is stable by linearization. The Jacobian matrix, evaluated at this equilibrium, is

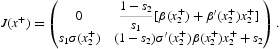

Note that 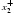, as a root of 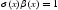, does not depend on *s*_2_ and that the eigenvalues of *J*(*x* ^+^) are λ_1_=1 and λ_2_=0 when *s*_2_=1. Define 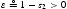 and treat the eigenvalues 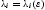 of *J*(*x* ^+^) as functions of 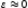. By continuity 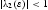 for 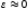. To determine the magnitude of 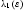 for 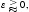 we calculate the derivative

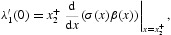

which, by Equation (13), is negative. It follows that 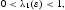 and hence A3(b) holds, for 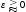.

From Theorem 3, we get the following result.

Theorem 4 Consider the juvenile–adult matrix model (1) with projection matrix (9) under the smoothness assumptions (10) on β and σ. Assume the backward bifurcation condition (11) and the inequality (13) hold. Then for 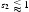 the juvenile–adult model has a strong Allee effect when 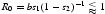.

We illustrate Theorem 4 with a specific example. This example will also illustrate other features of an Allee effect that can occur when created by a backward bifurcation. Take

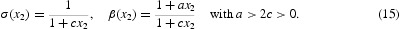

These functions satisfy the conditions required in Equation (10) with Ω taken to be the interval 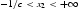. The backward bifurcation criterion (11)

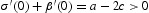

is satisfied.

What remains to be verified for the application of Theorem 4 is that the inequality (13) holds at a positive root of the equation 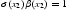, i.e. of the equation

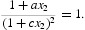

This equation has roots *x*_2_=0 and

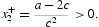

A calculation shows

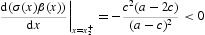

and hence inequality (13) does hold. Theorem 4 implies a strong Allee effect occurs in this example if 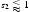 and 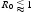.

For this example, we can in fact carry out further analysis. For positive equilibria, we can cancel *x*_2_ from the equilibrium equation (12b) and arrive at the equations

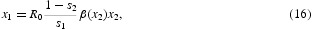

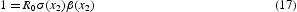

for positive equilibria. In the specific case (14), we can easily solve the second equation

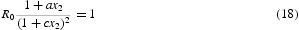

for positive solutions

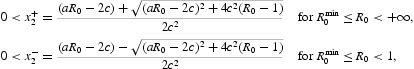

where

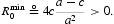

Note that

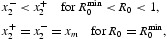

where we have defined

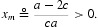

Together with

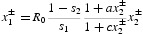

obtained from equilibrium equation (15a), these two roots define two positive equilibrium

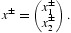

The spectrum 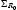 is the half line 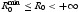 and there exist two positive equilibria *x* ^±^ for 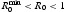 and one positive equilibrium *x* ^+^ for 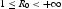. At *R*_0_=1, we have the (unique) positive equilibrium

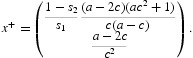



Having accounted for all positive equilibria in this example, we turn our attention to their stability properties. The Jacobian of the map 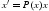 with projection matrix (9), when evaluated at an equilibrium pair (*R*_0_, *x* ^±^) is

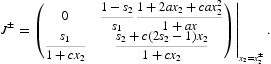

In calculating this Jacobian we made use of Equation (15). Stability by linearization is determined by the absolute value of the eigenvalues of *J* ^±^. The characteristic quadratic for *J* ^±^ is

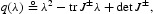

where

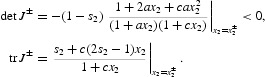

The quadratic *q*(λ) defines a concave upward parabola with a vertical intercept at det *J* ^±^<0. It follows that *q*(λ) two real roots

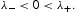

Simple geometric observations imply that

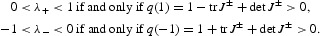

A calculation shows

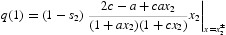

and therefore

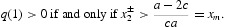

Since 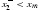 we conclude that *the equilibrium x* ^−^ *is unstable* (for all 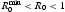). Since 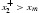 it follows that the stability (by linearization) of the equilibrium *x* ^+^ depends on λ_−_. Specifically,

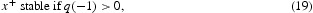

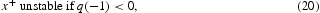

where

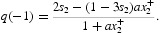

Using these stability/instability criteria, we distinguish three cases.

*Case* 1: If 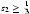 then the inequality (17a) holds and *x* ^+^ is (locally asymptotically) stable for all 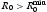. We conclude that *there is a strong Allee effect for all R*_0_ *satisfying* 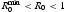.

Suppose *s*_2_<⅓. Since *q*(−1)=0 if and only if

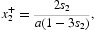

we see that

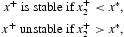

where

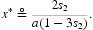

The equilibrium *x* ^+^ with component 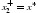 occurs for, and only for, 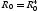 where

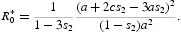

We conclude

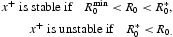



*Case* 2: Suppose

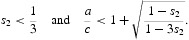

Some algebra shows that in this case 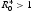. We again conclude that *there is a strong Allee effect for all R*_0_ *satisfying* 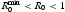.

Note that

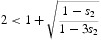

so that this inequality is compatible with *a*/*c*>2.

*Case* 3 Suppose

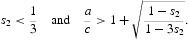

Some algebra shows that in this case 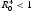. We conclude that for *R*_0_ satisfying 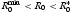 *there is a strong Allee effect*.

In Cases 2 and 3 the positive equilibrium's loss of stability as *R*_0_ increases through 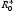 is the result of the eigenvalue λ_−_ decreasing through −1. This suggests a period doubling bifurcation of (stable) periodic 2-cycles. We will not formally prove that here, but the numerical simulation evidence (for example in Figures 2 and 3) corroborate this expectation. In Case 3 this means that *a strong Allee effect holds for R*_0_ *throughout the interval* 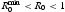, *but that it involves a positive equilibrium on the subinterval* 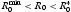 *and a stable 2-cycle on the subinterval* 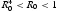.

**Figure 1.**
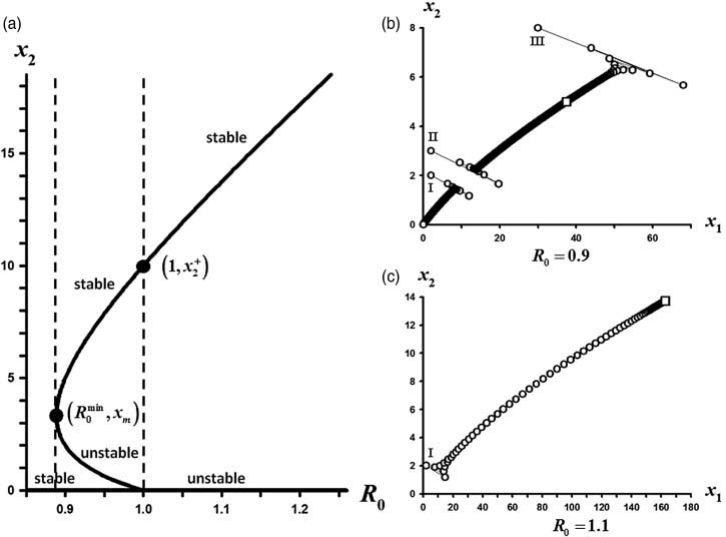
Parameter values are *s*_2_=0.5 and *s*_1_=0.1, *a*=0.3, *c*=0.1. (a) The *x*_2_ components of the positive equilibria from the bifurcating continuum *C* are shown plotted against *R*_0_. The two equilibria that mark the strong Allee interval (shown as solid circles) are 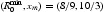 and 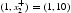. (b) For *R*_0_=0.9<1 a strong Allee effect is present. To illustrate this, three sample orbits in the phase plane are shown. The orbit with initial condition (*x*_1_, *x*_2_)=(2, 2), designated by I, tends to the extinction equilibrium while the orbits with initial conditions at (*x*_1_, *x*_2_)=(2, 3) and at (*x*_1_, *x*_2_)=(30, 8), designated by II and III, respectively, tend to the survival equilibrium (*x*_1_, *x*_2_)=(37.5, 5), indicated by the open square. (c) For *R*_0_=1.1>1 there is a unique positive equilibrium and it is stable. In particular, the orbit with initial condition (*x*_1_, *x*_2_)=(2, 2), designated by I, now tends to the survival equilibrium (*x*_1_, *x*_2_) ≈(162.88, 13.73), indicated by the open square.

**Figure 2.**
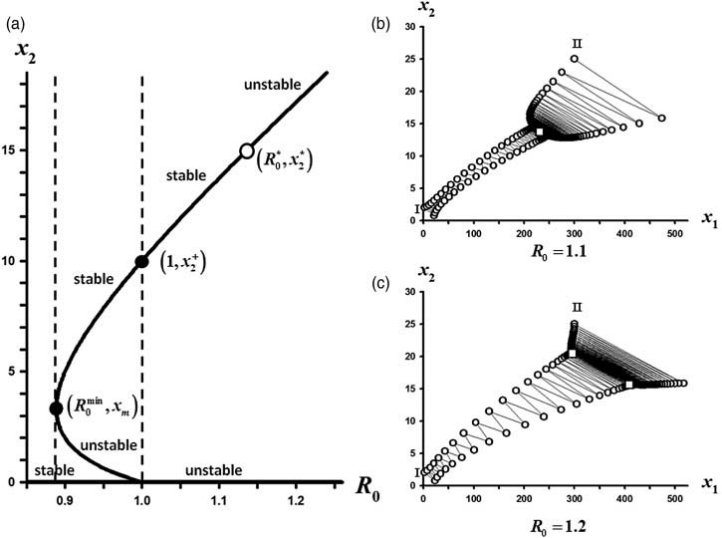
Parameter values are *s*_2_=0.29<⅓ and *s*_1_=0.1, *a*=0.3, *c*=0.1. (a) The *x*_2_ components of the positive equilibria from the bifurcating continuum *C* are shown plotted against *R*_0_. The two equilibria that mark the strong Allee interval (shown as solid circles) are 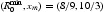 and 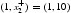. The open circle at 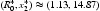 denotes where the positive equilibrium destabilizes. (b) For 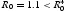 two sample orbits are shown. Orbit with initial conditions (*x*_1_, *x*_2_)=(2, 2) and (*x*_1_ *x*_2_)=(300, 25), designated by I and II, respectively, tend to the positive equilibrium (*x*_1_, *x*_2_)≈(231.29, 13.73), indicated by the open square. (c) For 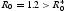 two sample orbits are shown whose initial conditions are the same as in (b). Both orbits now tend to a periodic 2-cycle whose points are (*x*_1_, *x*_2_) ≈(409.10, 15.66) and (*x*_1_, *x*_2_) ≈(296.22, 20.49), which are located at the open squares.

**Figure 3.**
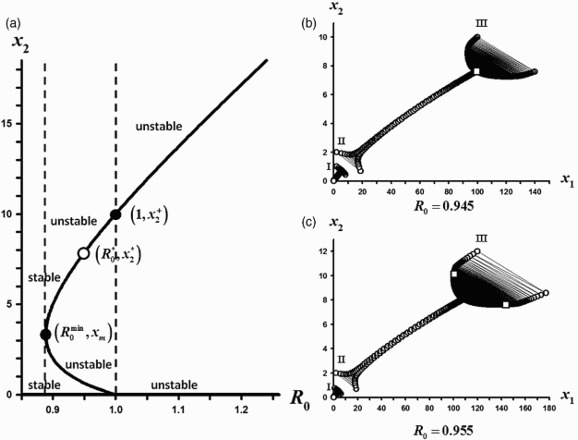
Parameter values are *s*_2_=0.26<⅓ and *s*_1_=0.1, *a*=0.3, *c*=0.1. (a) The *x*_2_ components of the positive equilibria from the bifurcating continuum *C* are shown plotted against *R*_0_. The two equilibria that mark the strong Allee interval (shown as solid circles) are 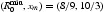 and 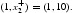 The open circle at 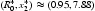 denotes where the positive equilibrium destabilizes. (b) For 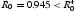 three sample orbits are shown. The orbit with initial condition (*x*_1_, *x*_2_)=(2, 1), designated by I, tends to the extinction equilibrium while the orbits with initial conditions at (*x*_1_, *x*_2_)=(2, 2) and at (*x*_1_, *x*_2_)=(100, 10), designated by II and III, respectively, tend to the positive equilibrium (*x*_1_, *x*_2_)=(99.53, 7.63), which is located at the open square. (c) For 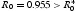 three sample orbits are shown. The orbit with initial condition (*x*_1_, *x*_2_)=(1, 0.75), designated by I, tends to the extinction equilibrium while the orbits with initial conditions at (*x*_1_, *x*_2_)=(2, 2) and at (*x*_1_, *x*_2_)=(120, 12), designated by II and III, respectively, tend to the survival periodic 2-cycle whose points are (*x*_1_, *x*_2_)≈ (143.80, 7.64) and (*x*_1_, *x*_2_) ≈(100.70, 10.14), which are located at the open squares.

The three scenarios represented by Cases 1–3 are illustrated in Figures 1–3. All three figures show the (parabolic) bifurcation diagram with the positive *x*_2_ component of the continuum of positive equilibria plotted as a function of *R*_0_. The strong Allee effect occurs in the region enclosed by the vertical dashed lines, caused by the backward bifurcation. Figure 1 illustrates Case 1 in which a strong Allee effect occurs throughout the interval 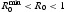 and a unique stable positive equilibrium exists for *R*_0_>1.

Figure 2 illustrates Case 2 in which a strong Allee effect occurs throughout the interval 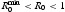, but in this case the unique positive equilibrium loses its stability as *R*_0_ increases above 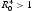, at which point stable 2-cycles appear as shown in the phase plane plots (b) and (c).

Finally, Figure 3 illustrates Case 3 in which a strong Allee effect occurs involving equilibria on the subinterval 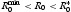 and involving a stable 2-cycle on the subinterval 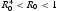.

## 5. Concluding remarks

We have seen that the interplay of positive feedback effects at low population densities (component Allee effects) and negative feedback effects at high population densities can together produce, in population matrix models, a strong Allee effect for an interval of inherent net reproductive numbers *r* (equivalently *R*_0_) with values less than 1. In general, strong Allee effects occur when there is a backward (transcritical) bifurcation at *r*=*R*_0_=1 and a saddle-node (blue-sky or tangent) bifurcation at a value of *r* (or *R*_0_) less than 1. This approach using bifurcation theory is different from that taken in [26] where different criteria for the occurrence of a strong Allee effect are obtained.

A strong Allee effect allows for the survival of a population when *r* (or *R*_0_) is less than 1, provided its density remains sufficiently high, i.e. does not lie in the basin of attraction of the extinction equilibrium (the Allee basin). The boundary of the Allee basin constitutes a threshold in phase space between extinction and survival. For *r*>1 (equivalently *R*_0_>1) the extinction equilibrium is unstable and a strong Allee effect cannot occur.

For a strong Allee effect to occur in this way, the component Allee (positive feedback) effects at low population density must be sufficiently large in magnitude, relative to any negative feedback terms (in the sense that κ defined by Equation (7) is positive), in order to produce a backward bifurcation of the bifurcating continuum 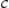 of positive equilibria. The negative feedback effects at high population densities cause the backward bifurcating continuum 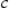 to ‘turn around’ or ‘fold over’ at a saddle-node bifurcation point, as illustrated in bifurcation diagrams in Figures 1(a)–3(a). It is this folding over of the continuum that accounts for the multiple positive equilibria for *r*<1 (equivalently *R*_0_<1) and, as a result, for the possibility of a strong Allee effect (should one of the positive equilibria be stable).

We focussed in this paper on strong Allee effects in which a stable extinction equilibrium coexists with a stable positive (survival) equilibrium. However, in the example in Section 4 we saw that a strong Allee effect can occur in matrix models in which a stable extinction equilibrium coexists with a positive attractor other than an equilibrium (namely, a stable 2-cycle).

Because the fundamental bifurcation Theorems 1 and 2 are the result of general bifurcation theorems and techniques from nonlinear functional analysis, analogs of these theorems have been proved for population dynamic models based on many other types of mathematical equations, including autonomous and periodically forced ordinary and partial differential equations, integro-differential equations and integro-difference equations. For some examples see [6–13,15,16,19,25]. The approach taken here towards strong Allee effects via backward bifurcations can also be taken for these types of model equations.

## Notes

1. A characteristic value of a matrix is the reciprocal of a real non-zero eigenvalue.

## Appendix 1. Proof of Theorem 1

We show that  by contradiction. If this set inclusion were not true, then since  is a continuum it would follow that  and we could choose a point  and a sequence  such that . Suppose first that  Then from  we find, by taking the limit as , that . Therefore *r**≠0 and since  it follows that . This is a contradiction to the Perron–Frobenius result (2b). Suppose, on the other hand, that *x**=0. Then from  we have, since  and hence *x*_*i*_≠0,

for all *i*. Take a subsequence, if necessary, so that the positive unit vectors  converge:  and . By taking the limit as  in Equation (A1) we see that  Since *u**≠0 it follows that *r**>0. Since  we have again contradicted the Perron–Frobenius result (2b).

The proof is complete when we have ruled out Rabinowitz alternative (ii). Suppose it were true that  meets (*rˆ*, 0) where *rˆ*≠1 is a real characteristic value of . Then there exists a sequence  such that . We have shown above that  are positive vectors and therefore Equation (A1) holds for this sequence. Extracting a convergent subsequence from the positive unit vectors  that converges to a unit vector  we have, by passing onto the limit, that  Since *rˆ*≠1 and *u** is a non-negative eigenvector of  we arrive at a contradiction to the Perron–Frobenius result (2b).

## Appendix 2. Proof of Theorem 3

Under assumptions A1 and A2 the trivial equilibrium is stable and there exists an unstable positive equilibrium for . Assumption A3 implies the existence of a positive equilibrium that is also stable. The proof of this follows from a straightforward application of the implicit function theorem to the equilibrium equation

centred on the pair (*r, x*)=(1, *x*_+_). The condition  in Assumption (A3) implies that the Jacobian of  evaluated at (*r, x*)=(1, *x*_+_) is non-singular. It follows that the equilibrium equation (A2) has a solution  for *r*≈1 that satisfies *x*(1)=*x*_+_. Since  and  it follows (by continuity) that  and  for *r*≈1. Therefore, the positive equilibria *x*(*r*) are (locally asymptotically) stable for *r*≈1.
